# Critical Limb Ischemia Due to Suspected Buerger’s Disease in an Adolescent Patient: A Case Report

**DOI:** 10.7759/cureus.58567

**Published:** 2024-04-18

**Authors:** Olga Silvestri, Angela Luongo, Alessandra Benenati, Davide Turchino, Paola Portella, Umberto Marcello Bracale

**Affiliations:** 1 Department of Public Health, University Hospital Federico II, Naples, ITA; 2 Department of Public Health/Anatomical Pathology, University Hospital Federico II, Naples, ITA

**Keywords:** critic limb ischemia, arteriopathy, vasculitis, buerger’s disease, thromboangiitis obliterans

## Abstract

Buerger’s disease is a segmental and inflammatory syndrome affecting relatively young individuals primarily and occurs with occlusion of small to medium-sized vessels in their extremities. The typical age for symptoms to appear is between 35 and 50 years in smoking patients. The disease is not manifested in children or the elderly. The only recognized successful treatment for it is immediate termination of smoking. In this report, we describe the case of a 16-year-old male suffering from this condition and referred from the pediatric department to our clinic, followed by a literature review of Buerger’s disease reported in adolescent patients.

## Introduction

Thromboangiitis obliterans (TAO), known as Buerger’s disease, is a segmental and inflammatory syndrome affecting mostly younger persons occurring with occlusion of small to medium-sized arteries and veins in their extremities [1]. The majority of patients affected are males who fit the typical symptom criteria: absence of ankle pulses, recurrent episodes of limb cooling, blanching, lower limb pain, and months/years of trophic disturbances leading to eventual gangrene and often requiring major limb amputation. The typical age at which symptoms appear is between 35 and 50 years in patients with a previous smoking history [2,3] and therefore the disease rarely develops in adolescents under 18 years old.

The diagnosis of Buerger's disease is based upon the same clinical criteria for the past 20 years, as defined by Shionoya et al. [4], which include (i) initial sign of symptoms prior to age 50, (ii) history of smoking, (iii) infra popliteal arterial occlusive lesions, (iv) upper limb involvement or thrombophlebitis migrant, and (v) lack of other atherosclerotic risk factors aside from smoking. The only recognized successful treatment is to immediately cease smoking although opinions on this matter are still divided [3,5,6]. However, as critical limb ischemia greatly lowers productivity and quality of life, revascularization, in addition to cessation of smoking, should be considered for patients with the condition [7].

## Case presentation

A 16-year-old male presented at our hospital’s pediatric emergency department with pain in the right lower extremity with the presence of right toe I-II ulcerations, fatigue, and cramp-like pain in the left calf. The patient was examined by the pediatric surgery department and treated with local wound debridement and courses of antibiotics without local improvement.

During outpatient evaluation, due to a worsening of his clinical condition, the patient was investigated for suspected vasculitis. His workup showed inflammatory indices, different autoantibodies, and plasmatic homocysteine within normal limits. The patient went through a molecular DNA study which showed factor V HR2 polymorphism, prothrombin G20210A polymorphism, and heterozygosity polymorphism for FV Hr2 (non-Leiden), indicating congenital thrombophilia, and therefore a predisposition for predominantly venous thromboembolic events. A microbubble test by the use of transcranial Doppler was also performed which showed permanent mild and latent grade shunt. The patient’s history was negative for other suspicious elements of autoimmune disease. 

During the outpatient evaluation, the patient underwent a vascular surgery consultation. Complete guided inquiries disclosed a five-month history of lower extremity discomfort, with ever-diminishing movement to offset and exacerbate the pain. His right foot and calves were the main locations of the pain, which was worsened by any physical activity including walking. The patient also reported color change of the extremities of the upper limbs when exposed to cold temperatures and admitted to smoking approximately 20 cigarettes a day during the past two years. He also confessed, in the absence of his mother, past use of unspecified narcotic substances, denying the use of cocaine. A drug screen was performed, which was negative. The 16-year-old patient suffered from a conduct disorder with an inability to limit his cigarette smoking despite the coercion of his mother and doctors. 

**Figure 1:**
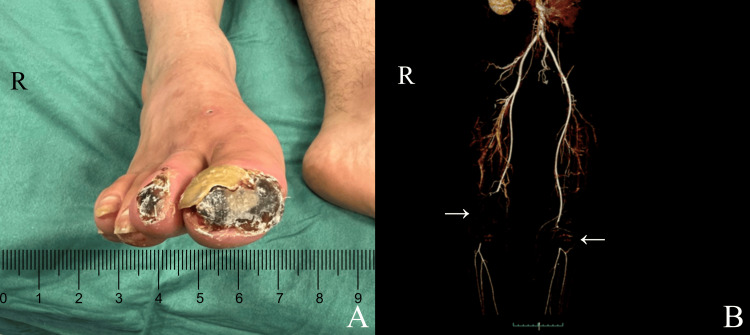
Ischemic ulcers in distal portion of right I-II toe (A). Posterior view of a three-dimensional reconstruction of a lower extremity contrast-enhanced CT arteriogram revealing obstruction of the right superficial femoral artery up to the level of the popliteal artery (A) as well as occlusion of the left popliteal artery (B).

A clinical examination revealed absence of distal peripheral pulses in the right lower limb. Also, the patient’s right side appeared to be colder with evidence of ischemic ulcerated lesions and gangrene of the I-II right toes (Figure 1).

A duplex scan, as well as a computed tomography angiography, showed occlusion of the superficial femoral artery from the middle-distal third of the thigh and reconstitution of the flow in the intraarticular popliteal artery of the right lower limb; the anterior tibial artery was not present, and the patient’s posterior tibial artery showed demodulated flow while in the left lower limb occlusion of the popliteal artery was noted.

Based on the previously mentioned data, our patient satisfied all Shionoya/Olin diagnostic requirements for TAO and fell into a Rutherford grade IV, Category 6 (clinical symptoms).

The patient's symptoms were first treated with oral analgesic therapy followed by epidural catheter, with continuous elastomeric pump infusion of 2 ml/hour of PGI2 analogue (Iloprost 0.05 mg/0.5 ml) simultaneously. Anticoagulation therapy commenced with unfractionated heparin (5000 IU twice daily) awaiting bypass surgery. A right femoral below-the-knee popliteal artery bypass was performed, using a reversed right great saphenous vein via medial approach. The young patient had an excellent recovery, beginning to walk in the unit by the next morning, and was discharged from the hospital three days later on acetylsalicylic acid (ASA) (100 mg once daily) and rivaroxaban (2.5 mg twice a day). 

**Figure 2:**
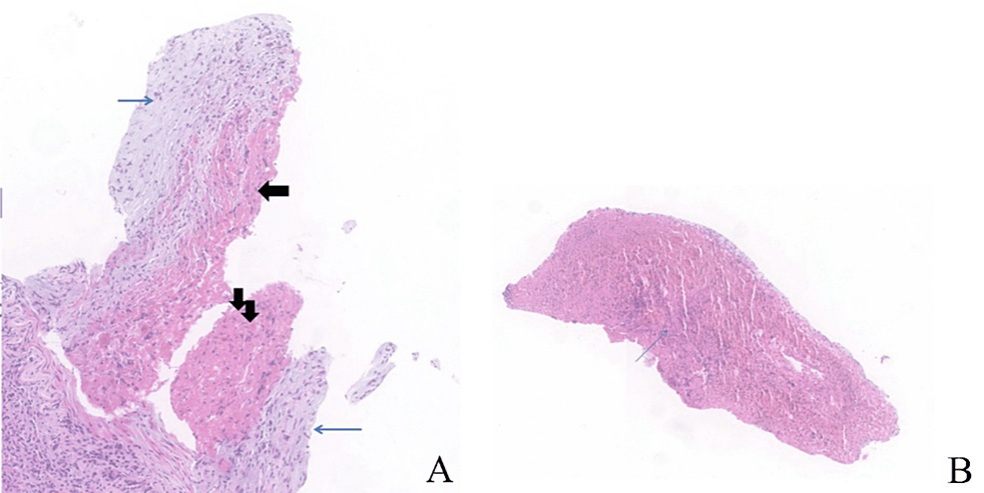
A low-power photomicrograph of a cross-section of the popliteal artery. Transmural inflammation is apparent without smooth muscle cell necrosis. No fragmentation of the elastic fibers: acute stage (A). A highly cellular thrombus, microabscess (blue arrow) are present within the thrombus: acute stage (B). (H&E 4X)

Popliteal artery specimens were gathered for histopathological analysis which revealed organized thrombus obstructing the artery's lumen. Lymphocytes and leukocytes were found in large numbers in the thrombus and the intima. No calcifications or atheromatous plaques were present in the vessel wall in any of the specimens. The tissue showed a necrotizing inflammatory appearance without atherosclerotic damage (Figure 2).

**Figure 3:**
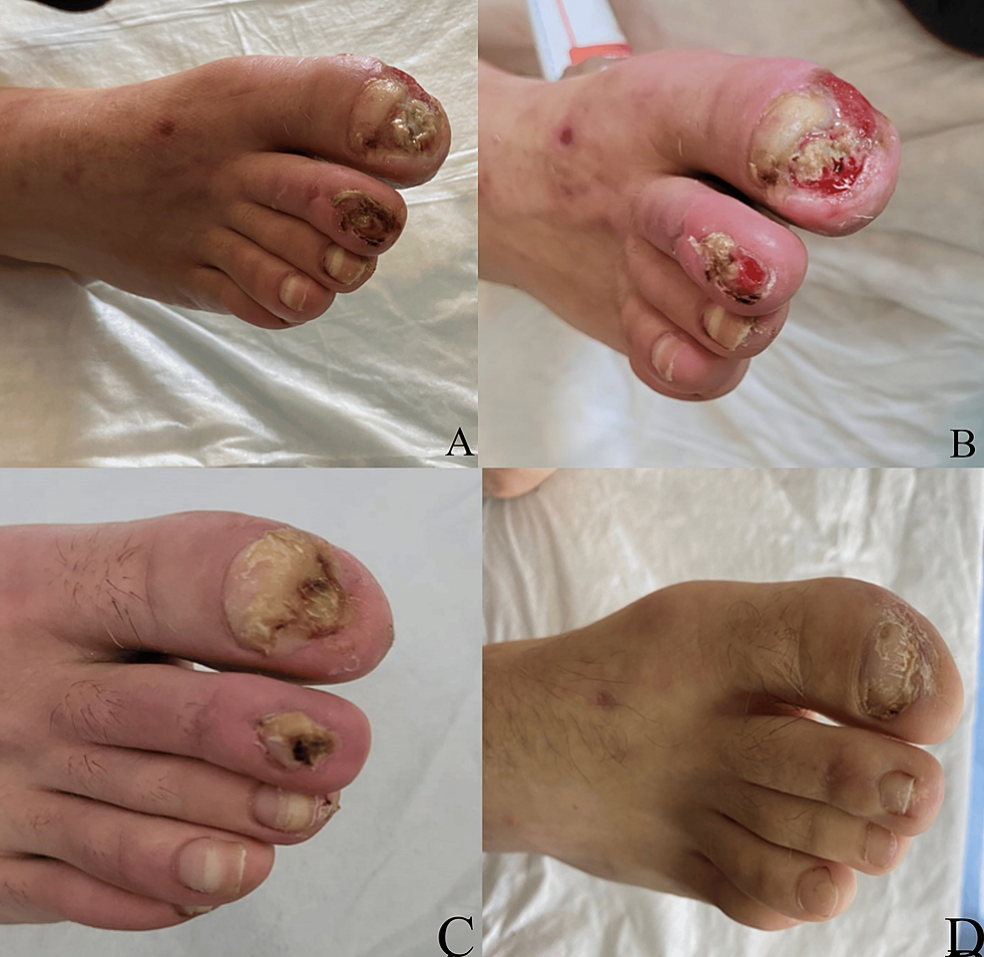
Progression of ischemic ulcers in distal portion of right I-II toe one week following surgical treatment (A-B). Progression of ischemic ulcers in distal portion of right I-II toe one and three months following surgical treatment (C-D).

At the clinical exam of the peripheral pulses, improvement was found after one week, at one month (Figure 3), and at six months. The Duplex scan on these occasions showed the patency of the bypass, consistent with the improvement of symptoms.

**Figure 4:**
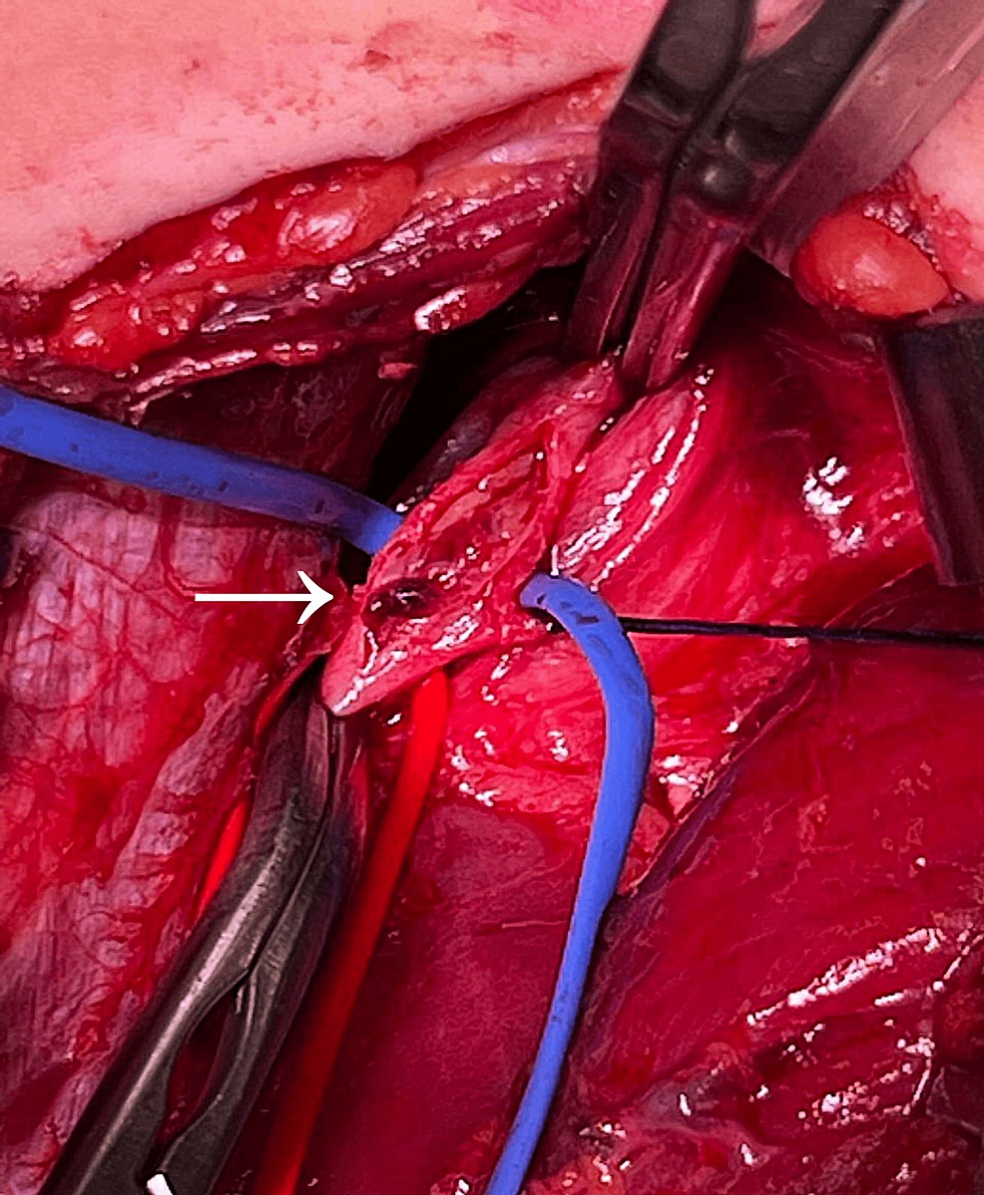
Popliteal artery lumen obstructed by organized thrombus.

The lesions of the first and second toes of the right foot were effectively improved, up to the closure and re-epithelialization. The popliteal arterial lesion biopsy at the time of bypass was in line with chronic-stage TAO (Figure 4).

## Discussion

TAO is an inflammatory vascular disorder that impacts the small and medium blood vessels in the upper and lower limbs and is unrelated to an atherosclerotic process. Neither children nor the elderly experience it and it is more common in men with a male-to-female ratio of 3:1. Tobacco and cannabis use are strongly linked [8,9] and the disease has become less common in the last 20 years as a result of lower smoking rates and stricter diagnostic criteria [10].

Through research on PubMed, using as filters "TAO", "Buerger's disease", and "adolescent or young adult patients", from 1950 to 2023, we finally selected five articles (Table 1). The analyzed articles showed that the average age of adolescents was 17.7 years old and the median monthly number of cigarettes smoked was 18.5 cigarettes [11-14]. One of the five patients from the reports reviewed, underwent leg amputation while the remaining four received a combination of medical and surgical therapy (Table 1).

**Table 1:** Literature review of thromboangiitis obliterans in adolescent patients

References	Age/sex	Clinical features	Site of disease	Smoking history	Therapy	Outcome
Laslett et al. [14]	17/F	Claudicatio pain/ Raynaud/Gangrene of right first toe	Right anterior tibial artery	10 pack years	Saphenous vein bypass, right lumbar sympathectomy, vasodilators, analgesic	No amputation
Matsushita et al. [12]	19/F	Claudicatio intermittens of left foot/Raynaud/ Gangrene of left foot and left middle finger	Infra-popliteal, radial and ulnar	3 pack years	Intra-arterial infusion of urokinase and heparin, serotonin antagonist, prostaglandin analogs, epidural anesthesia, warfarin	Below knee amputation of left leg
Lavie et al. [13]	19/M	Claudicatio intermittens in both lower limbs	Bilateral superficial femoral artery occlusion with corkscrew collaterals	10 pack years	Intra-arterial infusion of urokinase and heparin, prostaglandin analogs, percutaneous transluminal angioplasty, Clopidogrel/aspirin and enoxaparin	No amputation
Chapman et al. [11]	16/M	Claudicatio intermittens in both lower limbs/ Raynaud/ Gangrene of left first toe/Ulcers of second toe	Left superficial femoral artery occlusion	2 pack years + 7,5 gr. Cannabis daily	Femoral-tibial bypass/Dalteparin/Oppioids	No amputation
Bracale et al. (current case)	16/M	Claudicatio intermittens in both lower limbs/ Raynaud/ Gangrene of right first and second toe	occlusion of the right superficial femoral artery up to below the popliteal artery and occlusion of left popliteal artery	1 pack daily	Saphenous vein bypass, prostaglandin analogs, epidural anesthesia, anticoagulation with unfractionated heparin, Aspirin/Rivaroxaban (2,5 mg twice a day)	No amputation

Immediate and complete discontinuation of smoking seems to be the most effective method of TAO treatment. At the one-year follow-up in one of the biggest groups of TAO patients, a complete cessation of cigarette smoking resulted in zero amputations [15] and various medical treatments have had different degrees of success in the treatment of claudication pain [3-11]. Endovascular treatment of TAO is gaining attention as endovascular techniques progress [16,17]. It has been demonstrated to be effective; however data are still being compiled [18,19]. Endovascular therapy, according to reports, can be considered a realistic, safe, and successful treatment approach for TAO. 

## Conclusions

To the best of our knowledge, the 16-year-old patient described in the present case is probably among the youngest, if not the youngest, male patients treated for Buerger's disease. The disease is likely associated with a high consumption of tobacco and/or cannabis. At present, our patient is undergoing periodic checkups every six months. The trophic lesions have completely healed and a marked improvement is observed, although he has not completely ceased smoking. We continue to periodically evaluate our patient and his adherence to continuous therapy with ASA and rivaroxaban.
